# Dietary intake in lactating mothers in China 2018: report of a survey

**DOI:** 10.1186/s12937-020-00589-x

**Published:** 2020-07-14

**Authors:** Ye Ding, Wiwik Indayati, Til Bahadur Basnet, Fang Li, Hongliang Luo, Han Pan, Zhixu Wang

**Affiliations:** 1 Department of Maternal, Child and Adolescent Health, School of Public Health, Nanjing Medical University, Nanjing, Jiangsu 211166 People’s Republic of China; 2 Department of Epidemiology, School of Public Health, Nanjing Medical University, Nanjing, Jiangsu 211166 People’s Republic of China; 3 Danone Open Science Research Center for Life-transforming Nutrition, Shanghai, 201204 People’s Republic of China

**Keywords:** Lactating mothers, Dietary intake assessment, Food atlas, Online diary

## Abstract

**Background:**

The nutritional status of lactating mothers (LMs) is related to their own health and significantly impacts the secretion of breast-milk, and subsequently the growth and development of infants. Due to the influence of regional economy, traditional habits, and lack of nutrition knowledge, the problem of poor dietary nutrition among Chinese LMs is prominent. We aimed to evaluate and compare the dietary and nutrient intakes in LMs from urban and rural areas in China to provide baseline data for the implementation of relevant health guidance and strategies.

**Methods:**

A multi-stage sampling method was used to recruit urban and rural LMs from 13 provinces and municipalities in China. An online dietary record using food photographs was employed to keep track of what the LMs had eaten in 2 days in the form of face-to-face interview. A total of 954 participants were included in the final analysis. Data expressed as quartiles P50 (P25; P75) were compared using the Mann-Whitney U-test (level of significance: *p* < 0.05).

**Results:**

The consumption of staple food was higher in the rural (283.37 g/d) than in the urban areas (263.21 g/d). The consumption of vegetables, fruits, fish, shrimp, and shellfish, milk and dairy products was lower than the recommended amounts in both areas, and the insufficient intake of these food types was more serious in rural areas. While the energy intake of 83.8% of all LMs was lower than the estimated energy reference, it was comparable in the urban and rural areas. The intake of macronutrients (carbohydrates, protein, and fats) in rural areas was lower than in urban areas. The intake of some vitamins (VA, VB_1_, VB_2_, VB_9_ and VC) and minerals (calcium, magnesium, iodine and copper) was not ideal for LMs in both rural and urban areas.

**Conclusions:**

Overall, the dietary intake in LMs was lower than the recommended levels. Many essential nutrients failed to meet the recommended doses, both in the urban and rural areas. The deficiencies in micronutrients were more prevalent in rural compared to urban areas. Educating LMs about women’s health and appropriate dietary intake is, therefore, essential.

## Background

Lactating mothers (LMs) are more vulnerable to malnutrition compared to non-LMs worldwide. Nutrient intake by LMs is one of the most critical determinants of a woman’s health and well-being [1]. Poor nutrition during lactation places both mothers and growing children at a higher risk of disease, mental disorder, and death [2–4]. Therefore, this nutritionally high demanding state for a mother may cause a nutritive burden [5, 6]. Thus, LMs should increase their intake of energy (25%), protein (54%), and other nutrients [1, 2, 7, 8]. They should also be informed of healthy dietary practices and potential risks of nutrient deficiencies to achieve an optimal nutritional status during lactation.

The 2018 global nutrition report showed that China still experiences the burden of malnutrition among adult women. Approximately 26.4% of women of reproductive age have anemia, and 6.5% of them are obese [9]. Although the Chinese Nutrition Society (CNS) has established new guidelines to improve the nutritional status in Chinese women [10–12], the nutrient intake in this population remains below the recommended value. Some studies in China have reported that LMs had a significant lower intake of nutrients and did not reach the recommended levels for many essential micronutrients [1, 6, 13–15]. Eating habits of mothers, types of breastfeeding, and nutrition during lactation are contributing factors for the development of important endocrine, metabolic, and immunological disorders in infants [3]. The dietary intake status of LMs impacts the nutrient content of breast-milk and maternal health [1, 16, 17]. Breast milk is an ideal food for infants, which provides multiple nutrients and protection from diseases [1, 16, 18]. Therefore, mother’s diet needs to be well planned to ensure a sufficient supply of nutrients for infant’s health [5]. In 2016, the CNS added six recommendations to the “dietary guidelines for LMs,” based on the dietary guidelines for the general population. These include to (1) increase the consumption of animal-based foods including seafood, (2) select iodized table salt, (3) have a balanced diet, (4) get sufficient sleep, (5) perform moderate physical activity, and (6) avoid smoking and drinking concentrated tea or coffee [10].

Considering the importance of dietary assessment for LMs in China, a comparison of the nutritional intakes in urban and rural areas would help provide realistic dietary guidance based on the different areas. The conventional 24 h dietary recall method has been most commonly used to assess dietary intake. However, this method relies on the respondents’ descriptions to quantify their dietary intake during a specific period. Nevertheless, visual impression of the amount of food does not always correlate with its actual weight, making it difficult to estimate the food weight accurately. In recent years, using three visual reference systems, namely, regularly placed food portions, the two-dimensional background coordinates, and everyday objects known in daily life, we have developed a food atlas for dietary surveys in China. We found that, compared to the conventional 24 h dietary recall method, the estimate of food intake by the image quantification method was closer to the actual food weight obtained by weighing [19]. Therefore, we used the food atlas (image quantification) method to determine the dietary intakes in this study. The purpose of this study was to evaluate and compare the dietary and nutrient intake of LMs in urban and rural areas based on the diverse food habits and cultural groups in China.

## Methods

### Study participants

Our study was based on a dietary intake survey of LMs in China (2018–2019). A cross-sectional survey with a multi-stage sampling method was used to recruit participants from 13 provinces and municipalities locating in China. There are 31 provinces, autonomous regions, and municipalities in China, which are divided into seven regions. In the first stage, 13 provinces, autonomous regions, and municipalities (Shanghai, Beijing, Guangdong, Sichuan, Jiangsu, Fujian, Yunnan, Hebei, Hubei, Anhui, Liaoning, Zhejiang, and Henan) were chosen based on the geographical locations, economic status, and number of annual live births. In the second stage, each province, autonomous region, and municipality was divided into urban and rural areas, and then one survey city was selected for each area. In the third stage, study participants were randomly recruited based on the maternal information provided by the maternal and child health center in each city. Participants were requested for an interview via phone before conducting face to face interviews. Data collection was performed at a location agreed by both parties (usually at the participants’ home). The interviewers explained the purpose of the study and obtained signed informed consent from the women to participate in the study. Our study was approved by the Ethics Committee of Nanjing Medical University.

The study participants were healthy LMs aged 20–45 years with infants 0–24 months. Lactating mothers with metabolic diseases such as obesity, hypertension, and diabetes (including gestational diabetes), clinically diagnosed nutritional disorders (anemia, osteoporosis, iodine deficiency goiter, et al.), disability, mental disorder, infectious disease, any weakening or debilitating condition, or history of alcohol consumption, drug abuse, and smoking were excluded from the study. We also excluded participants who received medications (including herbal products, and traditional medicine) to treat a particular disease or medical condition, or were involved in other studies on food restriction.

The number of study participants was limited by the difficulty in assessing dietary intakes in China. We used Beaton’s formula to determine sample size, and the insoluble dietary fiber with high variability was used as parameter. It was calculated that 360 people were needed when the average nutrient intake per day within the 95% confidence interval and 10% deviation from the usual intake average. At first, 1113 LMs were selected from 13 provinces and municipalities for the study. Twenty six study participants were excluded due to no consumption of (1) cereal and their products, potatoes, and beans other than soybeans, and (2) vegetables, eggs, or any kind of meat (meat, fish and shrimp, poultry) in their daily meals. Then, 133 study participants were also excluded due to missing data and excessive dietary intake. Finally, from July 2018 to February 2019, 954 LMs (514 from urban and 440 from rural cities) were included in the study, which met our expectations.

### Dietary data collection and preparation

**Fig. 1:**
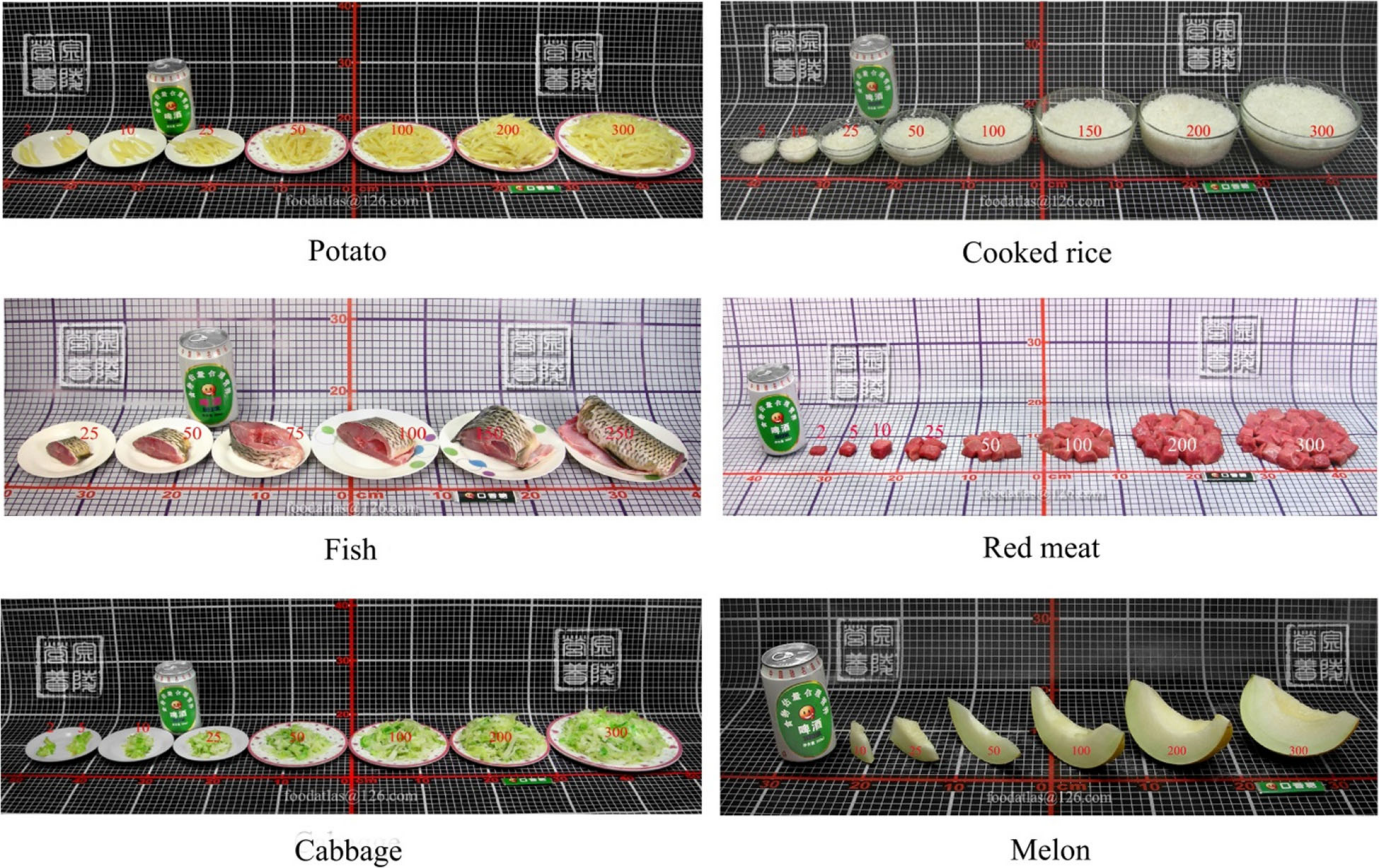
Food photographs (image quantification). Portion sizes from small to large (weight from light to heavy) with corresponding weight from 2 to 300 g of potato, cooked rice, fish, red meat, cabbage, and melon (adopted from food atlas) [19]

Dietary intake of LMs was assessed with the help of illustrated photos of food items (food atlas) [19] via an online diary with Tablet/iPad/Phone resources. Approximately 328 food/drink items were included in the food list [19, 20]. They were photographed against a standard background of board scales (Fig. 1). The board scale with 1 × 1 cm grid lines was used to increase the accuracy of the food record and to assist with the estimation of portion size [19]. All food items recorded were categorized into 14 groups based on the food group codes in the Chinese Food Composition Table [20]. Based on image quantification, food weight ranged from 2 to 300 g for the corresponding value of uncooked food. The foods were photographed in a round ceramic pan, plate, bowl, or cup [19].

The food list was integrated into two-day online diaries (weekend and weekday) to record food intake. Data were collected by third parties (Danone Open Science Research Center and Taylor Nelson Sofres). In the interview, well-trained interviewers asked participants to report all foods and beverages, including seasonings and supplements that they consumed on the weekend and weekday. For the intake record, meal times and descriptions of all foods, including beverages and supplements, were noted.

### Quality control

The questionnaire used in this survey was reviewed and revised by experts seven times to ensure its scientificity. A pilot study was conducted, and the data collected on anonymous samples were first reviewed to determine data quality. All investigators were trained on the technical aspects of the survey before conducting the study. Investigators on the spot helped the study participants with the online diet diaries. The responses were retrieved and reviewed in time. The errors were corrected to ensure the integrity and validity of the results.

### Dietary data analysis

The total daily intake of each kind of food was calculated, and the food had been in the common state, usually in the raw weight and 100% edible state. Particularly, in nine commonly consumed compound processed food, including dumplings, wonton, steamed stuffed buns, pizza, burger, sandwich, steamed vermicelli roll, zongzi (traditionally Chinese rice pudding), and tang-yuan (glutinous rice balls); we weighed every component in each of these foods (e.g., in a chicken burger, bread, vegetables, and chicken were considered individually) and calculated the ratio of individual components. Water intake was calculated from the total amount of drinking water and soup consumed. The food group intake recommendations for LMs based on the 2016 Chinese Balanced Dietary Pagoda [21] were used as a reference for setting up constraints for maximal quantities of each food group in the urban and rural areas.

The proportion of energy and nutrient content of each food were established from the Chinese Food Composition Table [20]. An excel worksheet was used for food conversion and calculation. Carbohydrates, fats, and proteins were converted from gram into kcal (1 g fat equals 9 kcal, and 1 g carbohydrate and protein equal 4 kcal) [22] to calculate the contribution of these macronutrients to the energy content of the food. Based on the 2013 Chinese Dietary Reference Intakes (DRIs), constraints were set up for energy and 25 nutrients [22]. The energy measurements were based on the estimated energy reference (EER) value. The lower limits of specific nutrients were based on adequate intake (AI) values. For some other nutrients, they were based on the estimated average reference (EAR) and recommended nutrient intake (RNI). The upper limits for the nutrients were based on the tolerable upper intake level (UL) values. Furthermore, for carbohydrates and fats, the acceptable macronutrient distribution ranges (AMDR) was applied as the lower and upper bounds for the percentage ratio of energy.

### Statistical analysis

Data were entered into an excel sheet from the two-day diet diaries, which were linked to the food items in the food list. The SPSS software package version 22.0 (IBM, New York, NY, USA) was used for statistical analysis. The median food intake from the two-day diet diaries was compared with the recommended daily food intake based on the Chinese Balanced Dietary Pagoda [21]. The nutrient intakes were compared with the Chinese DRIs [22]. The normality of the data was tested before analysis, and the data in non-normal distribution were expressed in quartiles; P50 (P25; P75). The Mann-Whitney U test was used to compare the differences between urban and rural areas. The number of study participants within and out of the intake recommendation ranges was calculated and expressed as N (%). Differences with a *P*-value < 0.05 were considered as statistically significant.

## Results

### Age of the study participants

A total of 954 LMs (mean age: 29.08 ± 4.033 years; range: 20–44 years) participated in the study. Table 1 shows the age distribution among LMs from the two areas. The mean age of the urban LMs (30.18 ± 4.026 years) was significantly higher than that of the rural LMs (27.99 ± 3.617 years).

**Table 1:** Age of lactating mothers living in urban and rural areas of China in 2018

Category	Total	Urban	Rural	*P*-value^1^
Sample Size	954	514	440	
Age (years), mean (±SD)	29.08 (±4.033)	30.18 (±4.026)	27.99 (±3.617)	0.000^*^
< 25, n (%)	180 (18.9)	59 (11.5)	121 (27.5)	
25–35, n (%)	699 (73.3)	395 (76.8)	304 (69.1)	
> 35, n (%)	75 (7.9)	60 (11.7)	15 (3.4)	
Lower, upper	20, 44	21, 44	20, 44	

^1^Significance level; ^*^p < 0.05 significant

### Consumption of foods from different categories

In the whole study population, cereals and their products, potatoes, and beans other than soybeans were the most popular staple food of daily consumption (Table 2). The consumption of these foods was markedly higher in the rural (283.37 g/d) compared to the urban areas (263.21 g/d). Among animal-based foods, the consumption of livestock meat and poultry (105 g/d) was higher compared to that of seafood (fish, shrimp, and shellfish) (10.05 g/d). Consumption of livestock meat, poultry, fish, shrimp, shellfish, and eggs was higher in the urban (200.81 g/d) compared to the rural areas (168.02 g/d). In rural areas, the intake of animal-based foods by LMs did not reach the recommended values. In general, approximately 55.2% of the respondents had lower than the recommended intake of livestock meat, poultry, fish, shrimp, shellfish, and eggs, while the stratified values for the urban and rural areas were 49.6 and 61.8%, respectively (Table 3).

**Table 2:** Food intakes in urban and rural lactating mothers from China in 2018

Food categories	Total (*n* = 954)	Urban (*n* = 514)	Rural (*n* = 440)	*P*-value^1^
Cereal and their products, potatoes and beans other than soybeans (g/d)	273.58 (201.43; 350.77)	263.21 (194.16; 340.93)	283.37 (212.84; 357.47)	0.007^*^
Potatoes (g/d)	19.7 (0; 50)	23.75 (0; 54.26)	12.5 (0; 49.25)	0.155 ns
Vegetables (g/d)	147.53 (78.59; 238.94)	177.36 (100.98; 265.12)	117.66 (61.96; 204.43)	0.000^*^
Green leafy vegetables and colored vegetables such as red and yellow (g/d)	147.57 (69.68; 246.71)	154.51 (72.55; 251.25)	139.35 (63.17; 238)	0.113 ns
Fruits (g/d)	69.05 (32.75; 122.96)	84 (42.65; 138.53)	53.75 (22.85; 100.25)	0.000^*^
Livestock meat, poultry, fish, shrimp, shellfish and egg (g/d)	184.18 (115.58; 280.61)	200.81 (139.23; 296.48)	168.02 (95.1; 257.22)	0.000^*^
Livestock meat and poultry (g/d)	105 (56.25; 179.72)	111.19 (64.03; 188.83)	98.89 (46.25; 166.32)	0.001^*^
Fish, shrimp and shellfish (g/d)	10.05 (0; 51.03)	14.5 (0; 57.79)	4.3 (0; 43.9)	0.010^*^
Egg (g/d)	45.59 (23.49; 69.6)	49.25 (27.38; 73.71)	34.31 (21.75; 64.38)	0.000^*^
Milk and its products (g/d)	100 (0; 207.5)	103.75 (0; 237.79)	68.4 (0; 192.91)	0.000^*^
Soybean and its products (g/d)	3 (0; 11.5)	4.2 (0; 13.31)	2.4 (0; 11.14)	0.065^*^
Nuts (g/d)	0 (0; 12)	0 (0; 11)	0 (0; 12.5)	0.571 ns
Cooking oil (g/d)	30 (25.65; 31.75)	30 (25.9; 32.33)	29.02 (25.5; 30.8)	0.000^*^
Water (ml/d)	1075 (650; 1625)	1050 (695; 1500)	1100 (600; 1800)	0.189 ns

The data have been presented as median (P_25_; P_75_)

^1^Statistically significant difference between urban and rural areas (*p* < 0.05) with Mann-Whitney U test; ns, *p* > 0.05 non-significant; ^*^p < 0.05 significant

**Table 3:** The recommended values and actual intake of foods in lactating mothers

Food categories	Total (*n* = 954)			Urban (*n* = 514)			Rural (*n* = 440)			Rec
	Below rec^a^	Above rec^b^	Rec^c^	Below rec^a^	Above rec^b^	Rec^c^	Below rec^a^	Above rec^b^	Rec^c^	
Cereal and their products, potatoes and beans other than soybeans (g/d)	579 (60.7)	241 (25.3)	134 (14)	326 (63.4)	120 (23.3)	68 (13.2)	253 (57.5)	121 (27.5)	66 (15)	300–350
Potatoes (g/d)	794 (83.2)	88 (9.2)	72 (7.5)	422 (82.1)	53 (10.3)	39 (7.6)	372 (84.5)	35 (8)	33 (7.5)	75–100
Vegetables (g/d)	886 (92.9)	35 (3.7)	33 (3.5)	471 (91.6)	23 (4.5)	20 (3.9)	415 (94.3)	12 (2.7)	13 (3)	400–500
Green leafy vegetables and colored vegetables such as red and yellow (g/d)	607 (63.6)	74 (7.8)	273 (28.6)	317 (61.7)	35 (6.8)	162 (31.5)	290 (65.9)	39 (8.9)	111 (25.2)	200–400
Fruits (g/d)	901 (94.4)	14 (1.5)	39 (4.1)	475 (92.4)	10 (1.9)	29 (5.6)	426 (96.8)	4 (0.9)	10 (2.3)	250–350
Livestock meat, poultry, fish, shrimp, shellfish and egg (g/d)	527 (55.2)	295 (30.9)	132 (13.8)	255 (49.6)	180 (35)	79 (15.4)	272 (61.8)	115 (26.1)	53 (12)	200–250
Livestock meat and poultry (g/d)	335 (35.1)	498 (52.2)	121 (12.7)	156 (30.4)	285 (55.4)	73 (14.2)	179 (40.7)	213 (48.4)	48 (10.9)	75–100
Fish, shrimp and shellfish (g/d)	788 (82.6)	111 (11.6)	55 (5.8)	416 (80.9)	67 (13)	31 (6)	372 (84.5)	44 (10)	24 (5.5)	75–100
Egg (g/d)	520 (54.5)	434 (45.5)	–	261 (50.8)	253 (49.2)	–	259 (58.9)	181 (41.1)	–	50
Milk and its products (g/d)	823 (86.3)	20 (2.1)	111 (11.6)	422 (82.1)	16 (3.1)	76 (14.8)	401 (91.1)	4 (0.9)	35 (8)	300–500
Soybean and its products (g/d)	854 (89.5)	100 (10.5)	–	456 (88.7)	58 (11.3)	–	398 (90.5)	42 (9.5)	–	25
Nuts (g/d)	699 (73.3)	255 (26.7)	–	384 (74.7)	130 (25.3)	–	315 (71.6)	125 (28.4)	–	10
Cooking oil (g/d)	175 (18.3)	373 (39.1)	406 (42.6)	82 (16)	233 (45.3)	199 (38.7)	93 (21.1)	140 (31.8)	207 (47)	25–30
Water (ml/d)	853 (89.4)	69 (7.2)	32 (3.4)	468 (91.1)	27 (5.3)	19 (3.7)	385 (87.5)	42 (9.5)	13 (3)	2100–2300

Shown is the comparison of the recommended values and actual intake of foods from different categories in lactating mothers from rural and urban areas of China in 2018

Data have been presented as number and percentage (n (%))

*Rec* recommended value

^a^Below rec: number and percentage of study participants whose food intakes are lower than the recommended value

^b^Above rec: number and percentage of study participants whose food intakes are higher than the recommended value

^c^Rec: number and percentage of study participants whose food intakes are within the recommended value

Furthermore, the consumption of plant-based foods, including fruits and vegetables (green leafy and colored) was higher in the urban than in the rural areas. Lactating mothers in both groups preferred vegetables over fruits. The consumption of vegetables and fruits in urban and rural areas was significantly different (*p* < 0.05) (Table 2). In both areas, the percent of LMs consuming less than the recommended amounts of vegetables and fruits was higher than those consuming more than the recommended quantity. The percentage intake of green leafy and colored vegetables (red and yellow) below recommendations was lower than other plant-based foods group in both rural and urban areas (Table 3).

We found that the consumption of milk and dairy products was lower than the recommended amounts in both areas. The median milk consumption in rural areas (68.4 g/d) was lower than that in urban areas (103.75 g/d) and the difference was statistically significant (Table 2). However, the consumption of cooking oil was high in both areas, with about 45.3% of urban LMs and 31.8% of rural LMs exceeding the recommended intake (Table 3). Due to the need for breastfeeding, LMs requires 2100–2300 ml of water per day; however, water intake in both groups did not reach the recommended value, and there was no significant difference between urban and rural areas (Table 2).

### Intake of energy, carbohydrates, protein, and fats

Table 4 shows the average daily intake of energy, carbohydrates, and fats. The energy intake was lower than the EER value in both groups. While the EER value for LMs is 2300 kcal/d, the median energy intake was 1711.12 kcal/d and 1576.34 kcal/d in the urban and rural areas, respectively. Approximately 18.9 and 13.2% of the LMs had an energy intake above the EER value in the urban and rural areas, respectively. The median carbohydrate intake was higher than the recommended amounts in both areas, and the middle quartile of LMs living in urban (64.6%) and rural (60.5%) areas met the recommended EAR value (above 160 g/d). The intake of insoluble dietary fiber was not ideal in both urban and rural areas (8.98 g/d vs 7.86 g/d), with 2.7 and 1.1% respondents from the urban and rural areas, respectively, consuming above the AI value (Table 6). The protein intake was slightly lower than the EAR value in both groups. While the EAR value for LMs is 70 g/d, the median protein intake was 66.58 g/d and 56.49 g/d in the urban and rural areas, respectively. Approximately 54.7 and 70.2% of the LMs had a protein intake below the EAR value in the urban and rural areas, respectively. Based on the AMDR values for fat, saturated fatty acid (SFA), monounsaturated fatty acid (MUFA), and polyunsaturated fatty acid (PUFA), we converted the AMDR values from percent of energy to grams per day. The median fat intake met the recommended value in both groups (Table 4**)**. In urban and rural areas, 44.6 and 42.7% of the LMs, respectively, had fat intake within the AMDR. Overall, while the consumption of carbohydrates was not significantly different between the two groups (*p* = 0.154), consumption of energy, protein, fat, SAF, MUFA, and PUFA were significantly different between urban and rural areas.

**Table 4:** Energy, carbohydrate, and fat intake in Chinese lactating mothers

	Total (*n* = 954)				Urban (*n* = 514)				Rural (*n* = 440)				*P*-value ^1^
	Median (P25; P75)	Below EER/EAR/AMDR^a^	Above EER/EAR/AMDR^b^	Within AMDR^c^	Median (P25; P75)	Below EER/EAR/AMDR^a^	Above EER/EAR/AMDR^b^	Within AMDR^c^	Median (P25; P75)	Below EER/EAR/AMDR^a^	Above EER/EAR/AMDR^b^	Within AMDR^c^	
Energy (kcal/d)	1640.95 (1327.45; 2085.97)	799 (83.8)	155 (16.2)	–	1711.12 (1377.34; 2174.62)	417 (81.1)	97 (18.9)	–	1576.34 (1272.82; 2013.97)	382 (86.8)	58 (13.2)	–	0.001^*^
Carbohydrate (g/d)	186.24 (134.77; 243.98)	356 (37.3)	598 (62.7)	–	191.75 (136.47; 251.42)	182 (35.4)	332 (64.6)	–	181.53 (133.71; 232.85)	174 (39.5)	266 (60.5)	–	0.154 ns
Fat (g/d)	72.68 (57.27; 90.12)	126 (13.2)	411 (43.1)	417 (43.7)	74.64 (59.87; 93.88)	51 (9.9)	234 (45.5)	229 (44.6)	70.53 (55.88; 87.3)	75 (17)^c^	177 (40.2)	188 (42.7)	0.002^*^
SFA (g/d)	16.88 (12.51; 22.2)	–	142 (14.9)	812 (85.1)	17.86 (13.25; 23.24)	–	91 (17.7)	423 (82.3)	15.71 (11.82; 21.4)	–	51 (11.6)	389 (88.4)	0.000^*^
MUFA (g/d)	31.37 (25.98; 37.46)	218 (22.9)	736 (77.1)	–	32.05 (26.95; 38.54)	95 (18.5)	419 (81.5)	–	30.24 (24.99; 36.71)	123 (28)	317 (72)	–	0.000^*^
PUFA (g/d)	13.53 (11.10; 17.29)	805 (84.4)	7 (0.7)	142 (14.9)	14.32 (11.55; 17.58)	433 (84.2)	3 (0.6)	78 (15.2)	12.86 (10.61; 16.66)	372 (84.5)	4 (0.9)	64 (14.5)	0.000^*^

Shown are the energy, carbohydrate, and fat intakes in lactating mothers from urban and rural areas of China in 2018

The data have been presented as number and percentage (n (%))

EER value is especially for energy, and the EER of lactating mothers was 2300 kcal/d

EAR value is for carbohydrates, and the EAR for lactating mothers was 160 g/d

AMDR values are for fat, SAF, MUFA, and PUFA, and these values in lactating mothers were 20–30%E, < 10%E, 25%E, and 7.6–15%E, respectively

%E is the percentage of energy provided by the nutrient as a percentage of total energy

^a^Below EER/EAR/AMDR: number and percentage of study participants whose intake of energy and nutrients are below EER/EAR/AMDR

^b^Above EER/EAR/AMDR: number and percentage of study participants whose intake of energy and nutrients are above EER/EAR/AMDR

^c^Within AMDR: number and percentage of study participants whose intake of energy and nutrients are within the AMDR

^1^Statistically significant difference between urban and rural areas (*p* < 0.05) with Mann-Whitney U test; ns, *p* > 0.05 non-significant; ^#^*p* < 0.05 significant

Table 5 shows the contribution of carbohydrate, fat and protein to the total energy intake. The median of contribution of carbohydrates to the total energy intake was comparable in the urban and rural groups (33.35% vs. 31.57%) with 52 (10.1%), and 45 (10.2%) of participants, respectively within the AMDR. However, energy derived from the intake of fats was predominantly above the AMDR for both groups. In addition, in urban areas, the percentage of LMs who were above the recommended AMDR of fat consumption was significantly higher compared to rural areas. The median of contribution of protein to the total energy intake was also comparable in urban and rural groups (8.64 and 14.68%, respectively).

**Table 5:** Energy ratios from carbohydrates, fats, and proteins in Chinese lactating mothers

Energy ratio (%)	Urban (*n* = 514)				Rural (*n* = 440)				AMDRs	*P*-value ^1^
	Median (P25; P75)	Below AMDR^a^	Above AMDR^b^	Within AMDR^c^	Median (P25; P75)	Below AMDR^a^	Above AMDR^b^	Within AMDR^c^		
Carbohydrate	33.35 (23.73; 43.73)	438 (85.2)	24 (4.7)	52 (10.1)	31.57 (23.25; 40.5)	379 (86.1)	16 (3.6)	45 (10.2)	50–65	0.651 ns
Fat	29.21 (23.43; 36.74)	51 (9.9)	234 (45.5)	229 (44.6)	27.6 (21.87; 34.16)	75 (17)	178 (40.5)	187 (42.5)	20–30	0.014^*^
Protein	8.64 (7.03; 10.23)	–	–	–	14.68 (11.79; 18.23)	–	–	–	ND	0.000^*^

Shown are the energy ratios derived from carbohydrates, fats, and proteins in lactating mothers from urban and rural areas of China in 2018

The data have been presented as number and percentage (n (%))

*ND* No AMDR value

^a^Below AMDR: number and percentage of study participants whose intake of energy ratio provided by macronutrients is below the AMDR

^b^Above AMDR: number and percentage of study participants whose intake of energy ratio provided by macronutrients is above the AMDR

^c^AMDR: number and percentage of study participants whose intake of energy ratio provided by macronutrients is within the AMDR

^1^Statistically significant difference between urban and rural areas (*p* < 0.05) with Mann-Whitney U test; ns, *p* > 0.05 non-significant; ^*^p < 0.05 significant

### Intake of vitamins and minerals

As shown in Table 6, except for VB_3_, VB_6_ and VB_12_, the intake of other vitamins (VA, VB_1_, VB_2_, VB_9_, VC and VE) was not ideal. The median of VA intake was comparable in the urban and rural groups (340.12 μg RAE/d vs. 251.12 μg RAE/d) with 475 (92.4%), and 427 (97.0%) of participants, below the EAR value in the urban and rural areas, respectively. The median of VB_1_ showed no significant difference between urban and rural areas. Approximately 59.3 and 58.4% of the LMs had the VB_1_ intake below the EAR value in the urban and rural areas, respectively. The VB_9_ intake of LMs in both rural and urban areas was at a low level. The number of participants below EAR value in the urban and rural areas was about 473 (92%) and 419 (95.2%), respectively. The median VC intake in rural areas (59.91 g/d) was lower than that in urban areas (81.09 g/d). In urban and rural areas, 71.0 and 78.9% of the LMs, respectively, had VC intake below EAR value.

**Table 6:** Intake of proteins, minerals, and vitamins in Chinese lactating mothers

		Total (*n* = 954)			Urban (*n* = 514)				Rural (*n* = 440)				*P*-value^1^
	Median (p25; p75)	Below EAR^a^	Above RNI/AI^b^	Above UL^c^	Median (p25; p75)	Below EAR^a^	Above RNI/AI^b^	Above UL^c^	Median (P25; p75)	Below EAR^a^	Above RNI/AI^b^	Above UL^c^	
Protein (g/d)	60.69 (47.84; 83.3)	590 (61.8)	269 (28.2)	–	66.58 (51.38; 88.36)	281 (54.7)	173 (33.7)	–	56.49 (45.03; 76.2)	309 (70.2)	96 (21.8)	–	0.000^*^
Insoluble dietary fiber (g/d)	8.47 (5.87; 12.24)	–	19 (2)	–	8.98 (6.31; 12.74)	–	14 (2.7)	–	7.86 (5.53; 11.83)	–	5 (1.1)	–	0.001^*^
VA (μgRAE/d)	302.31 (202.5; 454.9)	902 (94.5)	14 (1.5)	1 (0.1)	340.12 (234.71; 508.66)	475 (92.4)	11 (2.1)	–	251.12 (170.68; 402.5)	427 (97)	3 (0.7)	1 (0.2)	0.000^*^
VB_1_ (mg/d)	0.84 (0.5; 6.05)	562 (58.9)	366 (38.4)	–	0.86 (0.53; 5.92)	305 (59.3)	192 (37.4)	–	0.83 (0.49; 6.14)	257 (58.4)	174 (39.5)	–	0.574 ns
VB_2_ (mg/d)	0.85 (0.62; 1.27)	695 (72.9)	174 (18.2)	–	0.91 (0.67; 1.39)	353 (68.7)	113 (22)	–	0.78 (0.53; 1.13)	342 (77.7)	61 (13.9)	–	0.000^*^
VB_3_ (mgNE/d)	13.49 (9.99; 19.62)	374 (39.2)	255 (26.7)	33 (3.5)	14.93 (10.64; 20.97)	173 (33.7)	157 (30.5)	24 (4.7)	12.67 (9.43; 17.56)	201 (45.7)	98 (22.3)	9 (2)	0.006^*^
VB_6_ (mg/d)	1.83 (1.22; 2.7)	319 (33.4)	516 (54.1)	–	1.92 (1.31; 2.78)	149 (29)	300 (58.4)	–	1.67 (1.14; 2.6)	170 (38.6)	216 (49.1)	–	0.000^*^
VB_9_ (μgDFE/d)	212.27 (151.22; 298.16)	892 (93.5)	42 (4.4)	1 (0.1)	232.11 (165.41; 310.63)	473 (92)	31 (6)	–	194.71 (137.43; 279.78)	419 (95.2)	11 (2.5)	1 (0.2)	0.000^*^
VB_12_ (μg/d)	2.71 (1.57; 4.55)	451 (47.3)	384 (40.3)	–	2.83 (1.82; 5.04)	226 (44)	225 (43.8)	–	2.5 (1.4; 3.92)	225 (51.1)	159 (36.1)	–	0.000^*^
VC (mg/d)	70.29 (39.78; 126.32)	712 (74.6)	180 (18.9)	–	81.09 (46.99; 135.45)	365 (71)	111 (21.6)	–	59.91 (31.3; 109.43)	347 (78.9)	69 (15.7)	–	0.000^*^
VE (mg α-TE/d)	15.22 (15.22; 18.27)	–	408 (42.8)	–	15.22 (15.22; 18.27)	–	249 (48.4)	–	15.22 (15.22; 18.27)	–	159 (36.1)	–	0.000^*^
Calcium (mg/d)	412.57 (253.59; 647.79)	809 (84.8)	77 (8.1)	12 (1.3)	469.63 (287.62; 714.6)	417 (81.1)	53 (10.3)	9 (1.8)	358.38 (209.21; 560.16)	392 (89.1)	24 (5.5)	3 (0.7)	0.000^*^
Iron (mg/d)	18.46 (14.36; 24.44)	461 (48.3)	226 (23.7)	27 (2.8)	19.53 (15.07; 25.49)	218 (42.4)	143 (27.8)	13 (2.5)	17.24 (13.81; 22.79)	243 (55.2)	83 (18.9)	14 (3.2)	0.000^*^
Zinc (mg/d)	9.95 (7.27; 13.16)	473 (49.6)	310 (32.5)	–	10.43 (7.66; 13.52)	230 (44.7)	196 (38.1)	–	9.39 (6.99; 12.44)	243 (55.2)	114 (25.9)	–	0.001^*^
Magnesium (mg/d)	222.01 (162.80; 303.56)	667 (69.9)	188 (19.7)	–	236.61 (175.22; 312.8)	347 (67.5)	109 (21.2)	–	206.28 (149.28; 297.93)	320 (72.7)	79 (18)	–	0.000^*^
Potassium (mg/d)	3109.37 (2266.7; 4183.84)	–	683 (71.6)	–	2450.57 (1883.35; 3112.14)	–	269 (52.3)	–	4120.23 (3258.95; 5187.27)	–	414 (94.1)	–	0.000^*^
Phosphorus (mg/d)	802.06 (613.22; 1054.92)	217 (22.7)	583 (61.1)	–	852.73 (652.16; 1124.44)	89 (17.3)	349 (67.9)	–	748.31 (573.52; 982.36)	128 (29.1)	234 (53.2)	–	0.000^*^
Sodium (mg/d)	2885.46 (2745.6; 3102.64)	–	954 (100)	–	2783.77 (2686.97; 2902.93)	–	954 (100)	–	3070.91 (2889; 3301.59)	–	954 (100)	–	0.000^*^
Copper (mg/d)	2.64 (1.35; 10.71)	153 (16)	405 (42.5)	300 (31.4)	2.59 (1.36; 10.37)	75 (14.6)	230 (44.7)	153 (29.8)	2.73 (1.27; 11.07)	78 (17.7)	175 (39.8)	147 (33.4)	0.685 ns
Iodine (µg/d)	33.06 (18.38; 122.44)	744 (78)	89 (9.3)	105 (11)	34.95 (19.88; 136.65)	396 (77)	48 (9.3)	61 (11.9)	30.2 (17.19; 117.95)	348 (79.1)	41 (9.3)	44 (10)	0.160 ns

Shown are the intake of proteins, minerals, and vitamins in lactating mothers from urban and rural areas of China in 2018

The data have been presented as number and percentage (n (%))

RAE: retinol activity equivalent; NE: nicotinic acid equivalent; DFE: dietary folate equivalent; TE: tocopherol equivalent

The EAR of protein, VA, VB_1_, VB_2_, VB_3_, VB_6_, VB_9_, VB_12_, VC, calcium, iron, zinc, magnesium, phosphorus, copper and iodine were 70 g/d, 880 μgRAE/d, 1.2 mg/d, 1.2 mg/d,12 mgNE/d, 1.4 mg/d, 450 μgDEF/d, 2.6 μg/d, 125 mg/d, 810 mg/d, 18 mg/d, 9.9 mg/d, 280 mg/d, 600 mg/d, 1.1 mg/d and 170 µg/d

The RNI of protein, VA, VB_1_, VB_2_, VB_3_, VB_6_, VB_9_, VB_12_, VC, calcium, iron, zinc, magnesium, phosphorus, copper and iodine were 80 g/d, 1300 μgRAE/d, 1.8 mg/d, 1.5 mg/d, 18 mgNE/d, 1.7 mg/d, 500 μgDEF/d, 3.2 μg/d, 150 mg/d, 1000 mg/d, 24 mg/d, 12 mg/d, 330 mg/d, 720 mg/d, 1.4 mg/d and 240 µg/d

The AI of insoluble dietary fiber, VE, potassium and sodium were 25–30 g/d, 17 mg α-TE, 2400 mg/d and 1500 mg/d

The UL of VA, VB_3_, VB_6_, VB_9_, VC, VE, calcium, iron, zinc, phosphorus, copper and iodine were 3000 μgRAE/d, 35 mgNE/d, 60 mg/d, 1000 μgDEF/d, 2000 mg/d, 700 mg α-TE, 2000 mg/d, 42 mg/d, 40 mg/d, 3500 mg/d, 8 mg/d and 600 µg/d

^a^Below EAR: number and percentage of study participants whose intake of protein, minerals, and vitamins are below the EAR value

^b^Above RNI/AI: number and percentage of study participants whose intake of protein, minerals, and vitamins are above the RNI or AI value

^c^Above UL: number and percentage of study participants whose intake of protein, minerals, and vitamins are above the UL

^1^Statistically significant difference between urban and rural areas (*p* < 0.05) with Mann-Whitney U test; ns, *p* > 0.05 non-significant; ^*^p < 0.05 significant

Table 6 also summarizes the intake of minerals. The main problem with calcium, magnesium and iodine in LMs was inadequate intake, which was more serious in rural areas than in urban areas. The median intakes of calcium, magnesium and iodine in the total population were 412.57 mg/d, 222.01 mg/d and 33.06 µg/d, respectively. Although the intake of iron and zinc in rural areas was lower than that in urban areas, the dietary nutritional status of iron and zinc in LMs was acceptable. The median of iron and zinc in the total population was 18.46 mg/d and 9.95 mg/d. The dietary intake of potassium, phosphorus and sodium in LMs was relatively sufficient, but there were still differences between urban and rural areas. The intake level of potassium (4120.23 mg/d vs. 2450.57 mg/d) and sodium (3070.91 mg/d vs. 2783.77 mg/d) in rural areas was significantly higher than that in urban areas, but the intake level of phosphorus (748.31 mg/d vs. 852.73 mg/d) in rural areas was lower than that in urban areas. Different from the intake of other minerals, the excessive intake of copper in both urban and rural areas needs to be considered. The dietary copper intake of LMs from urban and rural areas exceeded UL value by 29.8 and 33.4% respectively.

## Discussion

Herein, we evaluated and compared the dietary and nutrient intakes in LMs from urban and rural areas in China. Quantitative pictures with a visual reference system were used to assist in the dietary survey, which was different from the conventional 24 h dietary recall method. Our findings indicated the need to improve the diet of LMs living in selected 13 provinces and municipalities (urban and rural), although the overall diversity in food consumption was comparable between the two areas.

In our study, we found that although urban women consumed more animal-based foods compared to rural women, the consumption of fish, shrimp, and shellfish did not meet the recommended values in both areas. In the rural areas, consumption of plant-based foods, including vegetables (green leafy and colored), and fruits were low. The consumption of these foods did not meet the Chinese recommended values in both areas. Similarly, both areas showed a low consumption of milk and dairy products, below the Chinese recommended values. Inferentially, LMs, especially in rural areas, should increase the consumption of animal- and plant-based foods to improve the quality of their diet. Limited food varieties and high prices were assumed to be the causes of the insufficient consumption in rural areas. Since Chinese people mainly rely on plant-based foods, especially in rural areas, they do not have the habit of drinking milk. Moreover, in Chinese culture, food taboos restrict the consumption of several foods during lactation, which also contributes to the imbalance in the diets of LMs.

The energy needs of LMs are increased because of breast-milk production [1, 2]; however, we found that of all the LMs from both areas who participated in this study, approximately 83.8% had lower energy intake than the EER value. Lactating mothers, especially in rural areas, should increase dietary intake to meet the lower limit of energy demand. Although the intake of carbohydrates was typically adequate in both urban and rural areas, the energy contribution from carbohydrates failed to meet the requirement, which is consistent with a previous study [5]. In addition, we also found that, the intake of insoluble dietary fiber was not optimistic in both areas due to excessive intake of fine processed food and insufficient intake of vegetables and fruits, which will bring about constipation, hemorrhoids and other health problems to LMs. Compared to non-LMs, LMs require approximately 54% more protein [2]; they should, therefore, consume more protein foods for optimal production of breast milk to promote infant growth, maintenance, and repair of cells [1, 6]. A study from southeast China found that LMs consumed about 31–53% more protein during the first 3 months of lactation than what was proposed by the Chinese RNI [1]. However, we found that the intake of animal-based food was lower than the recommended amounts. In particular, the consumption of fish, shrimp, shellfish and milk did not meet the recommended values. As a result, protein intake from animal-based foods also failed to reach the recommended value. The possible reason for this difference is that the time of our study is within 24 months after birth. As time goes on, LMs pay less attention to their diet.

A previous study has shown that fat intake in LMs was relatively high in Fujian, southeast China [1]. We found that fat intake, and its contribution to the total energy intake successfully met the required value set by the Chinese DRIs and was within 20–30% of the AMDR. A study on LMs in three cities (Beijing, Guangzhou, and Suzhou) has shown that fat/oil in the women’s diet influenced the fatty acid (FA) composition in breast milk [23]. Although fat plays an important role, whether a link between maternal diet and macronutrients in breast milk exists remains unclear [24]. Three studies from South Korea, Malaysia, and China have found that FAs in breast milk were positively associated with maternal FA intake [25–27]. Different types of FAs have different effects on the human body [24]; high intake of SFAs is a high-risk factor for cardiovascular disease [28], diabetes, coronary heart disease, stroke, and several types of cancer [1]. However, a high intake of MUFAs can improve serum lipid ratio [29], and that of PUFAs, including DHA, contributes in improvement in brain function and cognition [30]. Our study found that the intake of SFAs (82.3 and 88.4% in urban and rural areas, respectively) was within the recommended AMDR. Furthermore, the median intake of PUFAs met the required value, but a high percentage of LMs in both groups were below the AMDR. However, the intake of MUFAs was close to the DRI value.

Lactating mothers did not reach the Chinese RNI for all vitamins, except for urban LMs who did so for only VB_6_. A previous study of LMs in urban areas of China found that they were deficient in VA and VC intake [6], which were similar to our results. The source of VA is limited, mainly animal liver and dark-green leafy vegetables and fruits. If these kinds of food were not eaten at the time of investigation, the intake level of VA of LMs will be greatly different. The intake of animal liver once or twice a week should be encouraged in the diet of LMs. The intake of fruits and vegetables was lower than the required values, which also explains the lower intake of VC. Meanwhile, the lower intake of cereals, as well as beans and potatoes in the two survey areas, can explain the lower intake of some kinds of VB. Inadequate VB levels can affect the nutrient content of breast milk, thereby leading to growth retardation, anemia, anorexia, and neurological deficits in the infants [31]. The dietary intake of VB is firmly related to the area of residence and socioeconomic status of LMs. Previous studies reported that the intake of VB_1_, VB_2_, and VB_3_ were higher in the urban than in rural areas [31] and 72% of LMs had lower VB_9_ intake than the RNI value [1], which is consistent with our findings. Generally speaking, vegetable oil is the main food in China, and VE is relatively sufficient. In this study, the low level of VE may be related to the difficulty of estimation of dietary oil intake.

In the current study, a notable number of LMs in urban and rural areas failed to meet the EAR for iodine and magnesium and the RNI for iron and zinc. Nevertheless, all of them met the EAR values for phosphorus and copper and the AI for potassium and sodium. In agreement with our study, a previous study from Beijing, Suzhou, and Guangzhou has shown that intake of phosphorus and copper reached the recommended values in all LMs, while more than 50% of them had insufficient intake of iron, zinc, magnesium, and calcium [13]. Since breast milk contains approximately 38% iodine, meeting the iodine requirement during the lactation stage is critical for infants [32]. In view of the findings of our study, it is necessary to ensure that LMs eat seafood once or twice a week and use iodized salt for cooking. Meanwhile, our findings were consistent with those of previous studies reporting low calcium intake due to low consumption of milk during the lactation period [1, 5]. Overall, we found that the intake of key micronutrients essential during lactation, such as iron, calcium, iodine, and zinc was low and did not reach the Chinese recommendation for LMs, indicating the need for additional supplementation to improve the overall nutrient intake. Studies have shown that supplementation of multiple micronutrients improved the nutritional status among LMs [17, 23, 33].

In line with a previous study, our findings also suggested a conflict between the dietary recommendations and traditional practices in the community [5]. In agreement with a previous study [1], we also argue that single recommendations for a huge country like China are not feasible considering its geographical, cultural, and social-economic diversity. With a population of more than 1.34 billion people, China has 56 different ethnic groups and substantially different dietary patterns [1, 16]. The cultivation of complex food ingredients and eating habits may lead to an imbalance of nutrient intakes under specific conditions [34]. Chinese have a habit of preferring for their own kind of food; such as the northern Chinese prefer to eat more noodles or steam buns and hot spicy food, while the southern Chinese, in general, consume a considerable amount of rice, milk, and cold drinks [1]. It is, therefore, essential that the Balanced Dietary Pagoda and DRIs reflect the regional dietary habits and culture [21, 22]. Based on our findings and that of other studies in China, it would be critical to further track the health and disease risks for LMs and infants who do not receive adequate levels of nutrients. Insufficient nutritional intake directly affects maternal health, by reducing the amount of milk secretion and milk quality, thereby negatively impacting the growth and development of infants [35–37]. Accordingly, we recommend that LMs in China should be informed about the need for an appropriate diet to achieve and maintain optimal lactation without depleting mother’s nutrient reserves**.** Increased intake of fresh vegetables, fruits, fish as well as multivitamins and supplements are recommended for all women, especially for those who do not receive adequate intake of these foods and nutrients. Counseling for nutritional needs is also important for all women (non-LMs and LMs). Overall, a balanced diet is essential for the optimum health of mothers and infants.

However, our study has some limitations. First, we did not record the body weight and height of LMs, socioeconomic status, as well as anthropometrics of the infants as basic information for the study participants. Second, we used quantitative food atlas and a face-to-face dietary survey to help LMs recall the types of food they ate and improve the accuracy of food weight estimation. We also took quality control measures in the process of survey design, implementation, and data collection to minimize any bias. However, the dietary survey itself is very complex, coupled with the special group of LMs, who need postnatal recovery and taking care of infants. These conditions could have caused inevitable bias in data reporting. Third, we used only two-day diet diaries, due to limitation of the interviewers and LMs’ time. The unreasonable diet structures in some LMs might have been due to respondents misunderstanding the questions during the interview, as well as poor education on women’s health and diet. Finally, while we surveyed only healthy LMs, a separate study should focus on the underlying health conditions in those women.

## Conclusions

The diets of LMs in urban and rural areas in China are characterized by insufficient food and nutrient intake, which does not meet the Chinese Balanced Dietary Pagoda and DRIs. A nutritional imbalance was more evident among rural women compared to urban women. Based on our findings, we suggest more extensive dietary assessments in the future to understand the variations in the dietary structure of the population. We also hope that our findings will help in guiding policy changes to overcome malnutrition in LMs, especially in rural areas.

## Data Availability

The datasets generated and analyzed during the current study are not publicly available but are available from the corresponding author on reasonable request.

## Abbreviations

AI: Adequate intake
AMDR: Acceptable macronutrient distribution ranges
CNS: Chinese Nutrition Society
DRI: Dietary reference intake
EAR: Estimated average reference
EER: Estimated energy reference
FA: Fatty acid
LMs: Lactating mothers
MUFA: Monounsaturated fatty acid
PUFA: Polyunsaturated fatty acid
RNI: Recommended nutrient intake
SFA: Saturated fatty acid
UL: Upper intake level
